# Towards the Synthesis of Graphene Azide from Graphene Oxide

**DOI:** 10.3390/molecules201219747

**Published:** 2015-11-26

**Authors:** Christian E. Halbig, Philipp Rietsch, Siegfried Eigler

**Affiliations:** 1 Department of Chemistry and Pharmacy, Friedrich-Alexander Universität Erlangen-Nürnberg (FAU), Henkestraße 42, 91054 Erlangen, Germany; christian.e.halbig@fau.de (C.E.H.); philipp.rietsch@gmx.net (P.R.); 2 Institute of Advanced Materials and Processes (ZMP), Friedrich-Alexander Universität Erlangen-Nürnberg (FAU), Dr.-Mack-Straße 81, 90762 Fürth, Germany

**Keywords:** graphene oxide, graphene azide, Raman, TGA-MS

## Abstract

In the last decades, organic azides haven proven to be very useful precursors in organic chemistry, for example in 1,3-dipolar cycloaddition reactions (click-chemistry). Likewise, azides can be introduced into graphene oxide with an almost intact carbon framework, namely oxo-functionalized graphene (oxo-G_1_), which is a highly oxidized graphene derivative and a powerful precursor for graphene that is suitable for electronic devices. The synthesis of a graphene derivative with exclusively azide groups (graphene azide) is however still a challenge. In comparison also hydrogenated graphene, called graphene or halogenated graphene remain challenging to synthesize. A route to graphene azide would be the desoxygenation of azide functionalized oxo-G_1_. Here we show how treatment of azide functionalized oxo-G_1_ with HCl enlarges the π-system and removes strongly adsorbed water and some oxo-functional groups. This development reflects one step towards graphene azide.

## 1. Introduction

Graphene is a 2D nanomaterial consisting out of exclusively sp^2^ hybridized carbon atoms arranged in a honeycomb lattice. Intriguing properties like a high flexibility, transparency of 97.7% (visible regime) [1,2,3], charge carrier mobility values ranging from 10^3^ up to 10^6^ cm^2^/Vs [4,5] and a superior mechanical strength were discovered that originate from the honeycomb lattice [6,7,8,9]. In addition to pure graphene it can also be expected that novel properties can be discovered or engineered by the development of the chemistry on graphene. Therefore, the chemical modification is very promising for future medical and technical applications. Up to now, chemistry on the surface of graphene, edges and defect sites is just emerging. However, a complex task for the controlled chemistry of graphene results due to the inhomogeneity of natural graphite and related graphene [10,11,12,13,14,15]. The oxidation of graphite is known to yield graphene oxide in high yield, however lattice defects due to over-oxidation hamper the development of its controlled chemistry. In contrast, oxo-functionalized graphene (oxo-G_1_) bears defined amounts of lattice defects and they play therefore a minor role and surface chemistry is enabled. A typical density of lattice defects of about 0.3%–0.8% is achieved [14]. C-O bonds dominate the chemical motives in oxo-G_1_, but recently we succeeded in converting C-O bonds to C-N bonds by introducing the azide group [13]. We could show that organosulfate is substituted in solids by treatment of oxo-G_1_ with sodium azide and the major chemical functional groups of that derivative are hydroxyl and azide groups. Consequently, hydroxyl and azide functionalized graphene was produced with hydroxyl groups in majority. While graphene oxide is known for a long time, graphene decorated with exclusively azide groups is completely unknown. Also the synthesis of other “pure” derivatives, like graphane (fully hydrogenated graphene) [16,17] and graphene halides are still challenging [18,19]. Such structures, except graphane, would only bear an sp^2^-carbon lattice with only a single type of functional group attached to a sp^3^-carbon atom. It can be expected that graphene azide could act as a precursor for nitrogen doped graphene or functional graphene derivatives by introducing further addends.

**Scheme 1:**
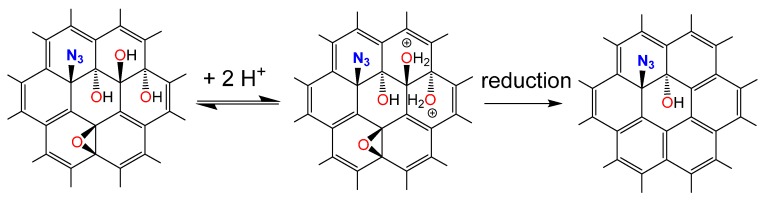
Proposed reaction leading to graphene azide with some additional hydroxyl groups.

Here we show how to functionalize single layers of oxo-G_1_ with azide groups (oxo-G_1_-N_3_) followed by HCl treatment. We observe an increased amount of aromatic C=C bonds and a significant removal of adsorbed water leading to a hydrophobic derivative of oxo-G_1_-N_3_. The product is analyzed by thermogravimetric analysis coupled with mass spectrometry (TGA-MS), UV-Vis, FTIR, Raman and elemental analysis (EA). Although hydroxyl groups are still present in HCl treated oxo-G_1_-N_3_, the expanded π-system is one further step towards the synthesis of graphene azide (Scheme 1).

## 2. Results and Discussion

**Figure 1:**
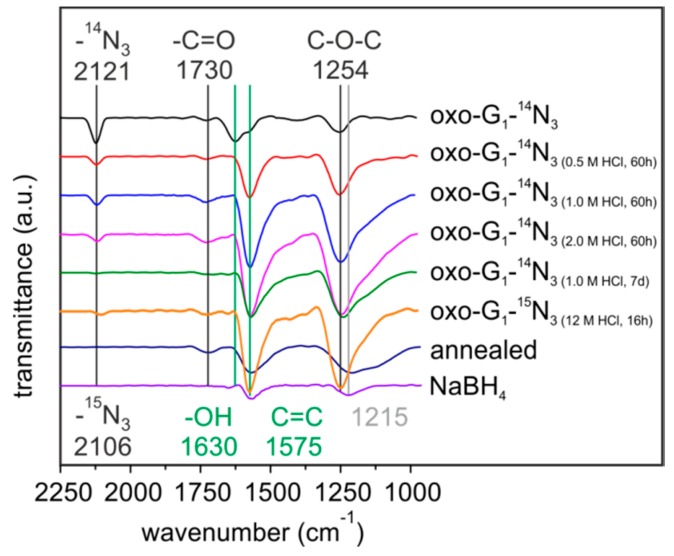
FTIR spectra of oxo-G_1_-^14^N_3_ and differently HCl-treated, annealed (140 °C) and sodium boron hydride reduced samples of oxo-G_1_-^14^N_3_.

**Figure 2:**
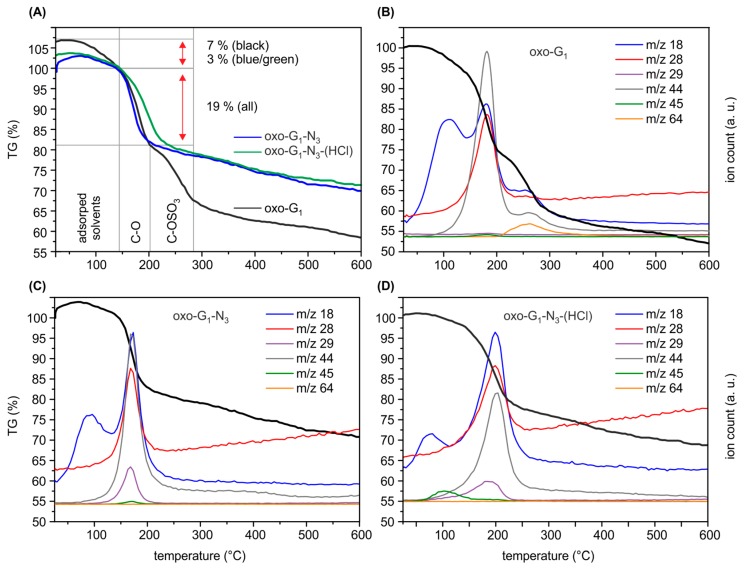
(**A**) Comparison of thermogravimetric analysis (TGA) results of oxo-G_1_ (black), oxo-G_1_-^15^N_3_ (green) and oxo-G_1_-^15^N_3_-(HCl) (blue); (**B**) TGA results of oxo-G_1_ with *m*/*z* traces of the most important peaks (*m*/*z* 18: H_2_O; *m*/*z* 28: CO_2_; *m*/*z* 29: ^15^N^14^N; *m*/*z* 44: CO_2_; *m*/*z* 45: H_3_CCOH^+^-fragment of 2-propanol and *m*/*z* 64: SO_2_-fragment from organosulfate); (**C**) TGA results of oxo-G_1_-^15^N_3_; (**D**) TGA results of oxo-G_1_-^15^N_3_-(HCl).

First, oxo-G_1_ was prepared by our previously introduced method [20]. Therefore, natural graphite crystals were oxidized in concentrated sulfuric acid with potassium permanganate at temperatures below 5 °C, followed by purification through repetitive centrifugation and redispersion in water. Delamination by tip sonication yielded single layers of oxo-G_1_ with hydroxyl-, epoxy- and organosulfate groups on both sides of the π-system. Carbonyl-, carboxyl- and ether groups are introduced at edges and in plane defect sites, but play a minor role due to the large flake size and the almost intact carbon lattice as determined by statistical Raman microscopy (SRM, defects < 0.3%–0.8%). Oxo-G_1_ was subsequently treated with sodium azide to obtain azide-functionalized oxo-G_1_. A nitrogen content of 3.3% was determined by EA and due to the carbon content of about 45% it can be calculated that one azide group is bound to around every 50th carbon atom (2% of N_3_, Table 1). Accompanied with introducing azide, organosulfate is cleaved and thus, the sulfur content decreases from 4.3% in oxo-G_1_ to 1.1% for oxo-G_1_-N_3_. Moreover the C-N_3_ bond formation can be directly probed by FTIR spectroscopy and the stretching vibration is detected at 2121 cm^−1^ (Figure 1). Also, TGA-MS supports that organosulfate was substituted by azide ions. As shown in Figure 2A the weight-loss step of organosulfate between 200 and 300 °C is only detectable for oxo-G_1_. This weight-loss step is accompanied with SO_2_ formation (*m*/*z* 64, Figure 2B) that originates from the reaction of organosulfate with the carbon grid. However, oxo-G_1_-N_3_ also bears hydroxyl groups and adsorbed water molecules. TGA-MS in Figure 2C reveals that water is released between room temperature and about 120 °C and 150 °C, detected by *m*/*z* 18 and a weight-loss of about 3%. Assuming that the elemental composition of oxo-G_1_-N_3_ contains in first approximation only C, H, N, S and O one can calculate a chemical formula of C_47.5_(OH)_28.8_(O)_5.7_N_3_(OH_2_)_2.1_(OSO_3_)_0.5_. Labelling of azide by ^15^N enables the detection of ^15^N^14^N by TGA-MS at *m*/*z* 29 (Figure 2C) [12,21]. While oxo-G_1_ forms a yellow-brownish dispersion in water oxo-G_1_-N_3_ is darker. This darkening comes along with an increased absorbance over the whole visible range spectrum and a shift of the absorption maximum from 234 nm to 237 nm.

**Table 1:** Elemental analysis of oxo-G_1_-^14^N_3_ and HCl treated derivatives.

Sample Name	C (%)	N (%)	H (%)	S (%)	O * (%)
oxo-G_1_	45.9	0.0	2.4	4.3	47.4
oxo-G_1_-N_3_	44.8	3.3	2.6	1.1	48.2
oxo-G_1_-N_3_-(2 M HCl)	54.8	4.5	2.6	0.8	37.3
oxo-G_1_-N_3_-(12 M HCl)	57.7	4.1	2.4	0.4	35.4

* O content calculated to meet 100%.

The addition of HCl_(aq)_ to oxo-G_1_-N_3_ leads to a decreased solubility depending on the reaction time and acid concentration (e.g., 0.5 M HCl treatment of 60 h at RT). Thus, structural changes must be responsible for that observation. The product was therefore isolated and thoroughly washed with water by centrifugation and redispersion in a 1/1 mixture of water and 2-propanol. Subsequent analyses via FTIR spectroscopy, EA, TGA-MS, UV-Vis and Raman reveal that graphene azide is not yet fully formed and hydroxyl groups are still present, but π-conjugated regions increased.

FTIR spectroscopy on aqueous HCl treated oxo-G_1_-N_3_ is depicted in Figure 1. For oxo-G_1_ FTIR features have been investigated since many years [22,23,24,25,26,27,28]. Regarding to the literature, peaks of oxo-G_1_ and oxo-G_1_-N_3_ can be related to the following functional groups: 3600–3000 cm^−1^ (C-OH); 2360* cm^−1^ (CO_2_); 2123/2112 cm^−1^ (^14^N_3_/^15^N_3_) 1730 cm^−1^ (C=O); 1630 cm^−1^ (adsorbed H_2_O); 1575 cm^−1^ (C=C); 1433 cm^−1^ (C-OH or carboxylate); 1254 cm^−1^ (C-O-C), 1060 cm^−1^ (C-O or C-C) [22,23,24,25,26,27,28]. After addition of diluted HCl the oxo-G_1_-N_3_ dispersion began to precipitate within minutes. A precipitate was immediately formed upon treatment with concentrated HCl. FTIR spectra were recorded on drop casted samples on ZnSe-windows (Figure 1).

Interestingly, the peak at 1575 cm^−1^ develops with increasing reaction time and acid concentration, whereas the peak at 1630 cm^−1^ declines and completely disappears (Figure 1). Since the signal at 1575 cm^−1^ is assigned to C=C bonds it can be assumed that the sp^2^ carbon network is growing. By the treatment of oxo-G_1_-N_3_ with HCl for 60 h, the FTIR spectra become less complex in the region between 1000 cm^−1^ and 2250 cm^−1^ and the spectra are very similar to NaBH_4_ treated oxo-G_1_-N_3_ and temperature annealed oxo-G_1_-N_3_ (140 °C), respectively. Moreover the absorption at 1215 cm^−1^ of reduced oxo-G_1_ is different compared to 1254 cm^−1^ for HCl treated oxo-G_1_-N_3_ (oxo-G_1_-N_3_-HCl). Importantly, the azide-peak (^14^N_3_: 2123 cm^−1^; ^15^N_3_: 2112 cm^−1^) is preserved upon treatment with diluted HCl (diluted 0.5 M–2 M for 60 h) and concentrated HCl treatment for very short reaction times (minutes to few hours). Long reaction times and higher acid concentration result both in cleavage of the azide group, e.g., 7 days treatment with 1 M HCl. Moreover the azide group is not stable upon treatment with a reducing agent, such as NaBH_4_, as well as temperature annealing [21].

**Figure 3:**
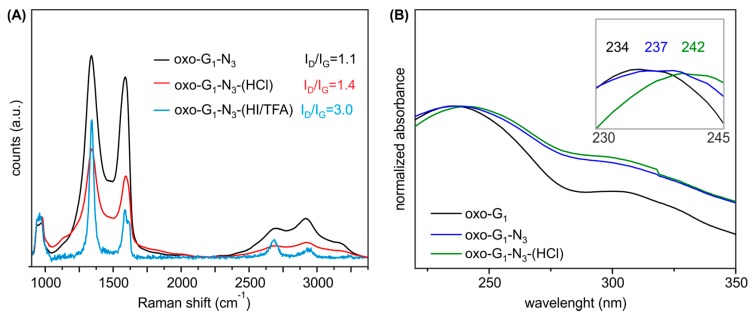
(**A**) UV-Vis spectra of oxo-G_1_ in water, oxo-G_1_-N_3_ and oxo-G_1_-N_3_-(HCl) in a 1/1 mixture of water/2-propanol; (**B**) Mean Raman spectra of films (on SiO_2_/Si wafer) of oxo-G_1_-N_3_, compared to oxo-G_1_-N_3_ treated with HCl and HI/TFA, respectively (measurement of the same area before and after treatment of oxo-G_1_-N_3_).

To clear up a potential loss of moieties, we further analyzed washed and freeze-dried oxo-G_1_-N_3_-(HCl) by EA (Table 1) and and TGA-MS (Figure 2D). EA indicates an increased carbon content for oxo-G_1_-N_3_-(HCl) (2M HCl treatment) of 54.8%, a decreased sulfur content to 0.4% and 4.5% nitrogen. Interestingly, the hydrogen content remained constant with 2.6%. TGA-MS reveals that the *m*/*z* 18 peak up to a temperature of 120 °C is smaller compared to oxo-G_1_ or oxo-G_1_-N_3_. This detection is in line with the observation that oxo-G_1_-N_3_ is almost not dispersible in water but in a mixture 1/1 mixture of 2-propanol. Also for purification, by means of centrifugation and redispersion, 2-propanol was necessary as an additional solvent. Consequently, the powder of isolated oxo-G_1_-N_3_-(HCl) (freeze-fried) still contains some 2-propanol, as indicated by the *m*/*z* 45 signal, that relates to the H_3_COH^+^ mass fragment of 2-propanol, with cleaved methyl radical (Figure 2D, green). Therefore, it is difficult to assign the 3% weight-loss up to 120–150 °C to a single solvent, despite water can also be cleaved from 2-propanol. Anyhow, since the hydrogen content in oxo-G_1_-N_3_-(HCl) is quite high, it seems likely that some 2-propanol is still present that consist of 1 oxygen and 8 hydrogen atoms. With that in mind we can calculate a molecular formula of C_42_(OH)_20.4_N_3_(OSO_3_)_0.2_(2-propanol)_0.5_. Within the experimental error, one can conclude that at least epoxy- and some hydroxyl groups were removed from the carbon lattice of oxo-G_1_-N_3_, leaving azide bound to the graphene lattice. A growing π-system upon HCl treatment of oxo-G_1_-N_3_ is also apparent by comparing UV-Vis spectra. As depicted in Figure 3B the absorption maximum shifts to 242 nm, what is another indication for the restoration of graphene patches.

Raman spectroscopy is a tool that developed to the major characterization technique for graphene materials and the ratio of the intensity of the defect induced D peak at about 1340 cm^−1^ to the G peak at about 1580 cm^−1^ can be used to determine the size of intact graphene patches [29,30,31]. As depicted in Figure 3A the intensity ratio of the D and G peak is roughly 1.1 for GO and the peaks appear very broad typical for oxo-G_1_ and GO in general. For comparison, oxo-G_1_ was chemically reduced by vapor of hydriodic acid and trifluoroacetic acid and the spectrum indicated graphene with a density of defects between roughly 0.3%–0.8% (I_D_/I_G_ ratio of 3.0) [29,30,32]. Raman spectra of oxo-G_1_-N_3_-(HCl), treated with 12M HCl, indicate the growth of graphene like patches by the increased I_D_/I_G_ from 1.1 to 1.4. By measuring unreduced and so highly functionalized oxo-G_1_-N_3_-(HCl) an increase of I_D_/I_G_ ratio indicates a reduction of defect sites as we are in the high defect regime [29,32].

## 3. Materials and Methods

### 3.1. Chemicals and Instrumentation

Graphite grade 3061 was purchased from Asbury carbon mills (Asbury, NJ, USA). Potassium permanganate, sulfuric acid, hydrogen peroxide, sodium azide and hydrogen chloride solution were purchased from Sigma Aldrich^®^ (St. Louis, MO, USA) and double distilled water from Carl Roth^®^ (Karlsruhe, Germany). A Sigma 4K15 centrifuge was used with 200 mL plastic beakers. Transmission FTIR spectra were recorded on a Bruker Tensor27 (Bruker, Billerica, MA, USA) and ZnSe-windows had been used. TGA-MS was performed on a Netzsch Skimmer STA 409 CD coupled with mass spectroscopy (NETZSCH-Gruppe, Selb, Germany). Raman spectra were recorded with the confocal Raman spectrometer LabRAM Aramis (Horiba, Kyoto-Shi, Japan) equipped with a second harmonic 532 nm Nd-YAG laser for excitation. The acquisition was set to 0.2 s. The absorbance of our samples was recorded using quartz cuvettes and a Lambda 1050 (Perking-Elmer, Waltham, MA, USA) in the wavelength from 200 to 600 nm.

### 3.2. Synthesis of Oxo-G_1_ and Oxo-G_1_-N_3_

Dry oxo-G_1_ and oxo-G_1_-N_3_ powder were synthesized as described in the literature [20]. Graphite crystals (1 g) were mixed with 24 mL of sulfuric acid (98%) for 1 h followed by the slowly addition of potassium permanganate (2 g) over 4 h. The mixture was further stirred for 16 h. After the stepwise dilution of the reaction mixture with diluted sulfuric acid (20%, 20 mL, 4 h) and pure water (100 mL, 16 h) we added aqueous hydrogen peroxide (5%, 40 mL, 40 min) and centrifuged for 6 times (1500 RCF, 10 min). This mixture was delaminated with by tip sonication for 4 min at 20 W with a flat titan tip. The obtained dispersion was again centrifuged for a few times to remove non-monolayer particles (RCF < 1500, 30 min) and smallest nano-particles (13,000 RCF, 45 min). We obtained a stable, yellow-brownish oxo-G_1_ dispersion with main flake sizes of 1 μm to 20 μm. The concentration of 0.488 mg/mL was calculated by freeze-drying a small amount of the dispersion.

To obtain oxo-G_1_-N_3_, the dispersion containing 100 mg oxo-G_1_ was stirred with 80 mg of Na^14^N_3_ for 1 h followed by freeze-drying. Purification was accomplished by repetitive centrifugation (3 times, 13,000 RCF, 45 min). The yield after lyophilization was 77.6 mg [13]. Oxo-G_1_-^15^N_3_ was produced similarly, but on half scale (yield: 38.8 mg).

### 3.3. Preparation of Oxo-G_1_-N_3_-(HCl)

All dried samples had been dispersed in pure water to obtain dispersions with a concentration of 1 mg/mL (10 mg, 10 mL) before the treatment with HCl_(aq)_. To this, 10 mL of differently concentrated HCl solutions (0.5 M; 1.0 M; 2.0 M; 12 M) were added. The samples were centrifuged for purification (3 times, 13,000 RCF, 45 min) and redispersed with water or 50:50 water/isopropanol.

To obtain a temperature annealed oxo-G_1_-N_3_ samples, few drops of the dispersion had been dropped onto a ZnSe window and put onto a heated magnetic stirrer to obtain a temperature annealed sample (140 °C, 10 s). For a chemical reduced reference, a few mL of the dispersion (10 mg oxo-G_1_-N_3_) were treated with a small excess of sodium borhydride (16 mg), purified by repetitive centrifugation and dropped on ZnSe windows after redispersion.

## 4. Conclusions

We observed changes of the physical properties of HCl treated oxo-G_1_-N_3_. FTIR spectroscopy confirmed an enlarging of aromatic areas due to assumed rearrangement and reduction. A reduction is plausible if chloride can be oxidized by protonated oxo-G_1_-N_3_. Regular reduction protocols of oxo-G_1_-N_3_ samples as for examples NaBH_4_ or HI/TFA lead to an unselective cleavage of all surface moieties resulting in graphene [14,33]. Therefore, the here presented investigation preserves most of azide groups on the carbon lattice, while oxo-functional groups are removed. However, the HCl treatment of oxo-G_1_-N_3_ does not lead to a complete cleavage of oxygen containing moieties, but at least the π-system enlarged as evidenced by TGA-MS, EA, Raman, FTIR and UV/Vis. The regiochemistry of hydroxyl groups and azide groups remains, however, speculative. Thus, the work presented here represents one step towards the synthesis of graphene azide, a derivative of graphene that stands in line with graphene or halogenated graphene.
